# Acceptability of Teledentistry in Mobile Oral Health Applications for Australian Children: A Qualitative Study

**DOI:** 10.1002/hpja.70280

**Published:** 2026-11-04

**Authors:** Johanna Groundwater, Ria Aiyar, Lisa Jamieson, Tamanna Tiwari, Estie Kruger, Marc Tennant, Sonia Nath

**Affiliations:** ^1^ School of Dentistry Adelaide University Adelaide Australia; ^2^ School of Dentistry University of Minnesota Minneapolis Minnesota USA; ^3^ School of Health and Clinical Sciences The University of Western Australia Perth Australia

**Keywords:** mHealth, oral health education, paediatric dentistry, qualitative research, teledentistry

## Abstract

**Issues Addressed:**

Access to timely dental care remains inequitable for children in rural, remote and disadvantaged communities. Teledentistry could narrow this gap, and mobile oral health apps offer a platform, yet user acceptance remains largely unexamined. This study explored how parents, public health professionals, software and design professionals and dental professionals perceive a teledentistry feature within a children's oral health app, and the equity and governance issues it raises.

**Methods:**

Parents and caregivers, public health professionals, software and design professionals and dental professionals were recruited through purposive and snowball sampling via professional networks, social media and organisational contacts. Participants completed an online questionnaire capturing demographic characteristics and perceptions of mobile oral health technologies, followed by a semi‐structured interview conducted in person, by telephone or via videoconference by one of four researchers. Interviews were audio‐recorded and transcribed verbatim. Questionnaire data were summarised descriptively, and interviews were analysed using Braun and Clarke's six‐phase reflexive thematic analysis. Two researchers coded the data and developed preliminary themes.

**Results:**

A total of 59 participants (39 women, 20 men) were interviewed, and four themes were developed: (1) acceptance of a teledentistry feature within a mobile oral health application; (2) the potential for teledentistry to improve access and equity; (3) preferences for in‐person care, reflecting concerns about diagnostic accuracy, communication and clinician–patient relationships; and (4) perceived logistical and governance challenges to implementation. Participants viewed teledentistry as an acceptable, complementary feature for screening, triage, follow‐up and continuity of care, recognising its potential to reduce geographical, social and systemic barriers, particularly for rural and isolated paediatric populations. However, concerns persisted that diagnostic accuracy, communication, trust and cultural safety are difficult to replicate remotely, alongside logistical and governance challenges including cost, funding, workforce capacity, oversight and safeguarding.

**Conclusions:**

Teledentistry has the potential to improve equitable access to oral healthcare for children and families. However, its effectiveness depends on careful design and on its recognition as a trusted supplement to, rather than a replacement for, in‐person care. This approach requires strong governance, sustainable funding and child‐safe consent.

**So What?:**

Teledentistry is likely to be accepted by families and professionals only when it is positioned as an adjunct that extends, rather than substitutes for, in‐person paediatric dental care. Developers and policymakers should therefore prioritise triage, follow‐up and continuity functions, with clear pathways to a clinician and attention to cultural safety.

## 1 Introduction

Globally, teledentistry is increasingly recognised as an effective way to expand access to oral healthcare, especially for underserved populations, by overcoming barriers related to geography, cost, social factors and workforce limitations that often restrict traditional dental services [1, 2, 3]. In Australia, the implementation of teledentistry is guided by professional standards developed by the Australian Dental Association, which outline requirements for informed consent, patient privacy and confidentiality, record‐keeping, clinical governance and the appropriate use of digital communication technologies in the delivery of oral healthcare [4]. A growing body of evidence demonstrates that teledentistry can support caries screening, diagnosis and preventive care in paediatric populations across diverse settings, with diagnostic accuracy comparable to conventional visual examination when supported by appropriate imaging and trained personnel [5, 6].

Teledentistry, defined as the delivery of oral healthcare services and information through digital communication technologies, can be delivered synchronously (real‐time), such as videoconferencing and telephone consultations, or asynchronously (store‐and‐forward), whereby clinical data, digital images and radiographs are collected and transmitted electronically for subsequent review by oral health practitioners [1]. Both models are well suited to paediatric care, where a parent can capture images of a child's teeth for remote review or join a real‐time consultation on the child's behalf. In Australia, paediatric teledentistry models have shown particular promise; a study of schoolchildren aged 4–15 years in Western Australia evaluated a teledental pathway against routine care [7], and a subsequent feasibility study demonstrated that parent‐operated, app‐based tele‐dental screening could reliably identify early childhood caries in preschoolers [8]. Together, these studies indicate that mobile, parent‐mediated teledentistry is both feasible and well suited to the realities of caring for children's oral health.

Despite the growing adoption of teledentistry and mobile oral health technologies, evidence on how the people who use, deliver and design them perceive their integration into children's oral healthcare remains limited and fragmented. What is known has largely developed along two separate strands and within single stakeholder groups. In relation to teledentistry, parents report high acceptance of remote paediatric consultations for triage, follow‐up and continuity of care, while remaining cautious about complex presentations that warrant in‐person examination [9]; dental professionals, in turn, hold divergent views, weighing improved access against concerns about diagnostic quality, upfront cost, workflow and reimbursement [10]. Regarding mobile applications, end users such as children and adolescents find oral health education apps acceptable and engaging, yet these tools are typically evaluated as standalone educational aids rather than as gateways to clinical care [11]. Stakeholders' expectations, and the barriers and facilitators they anticipate, remain poorly characterised in the current literature, yet are central to ensuring that these technologies are clinically appropriate, user‐friendly and aligned with both evidence‐based standards and community needs. Therefore, this study aims to explore the perceptions, experiences and attitudes of parents, dental professionals, software/design professionals and public health professionals regarding the use and potential implementation of teledentistry as a feature in mobile oral health applications for children's education and prevention.

## 2 Methods

### 2.1 Study Design

The reported data are part of a broader study evaluating the perceptions and experiences of mobile oral health applications for Australian children. Ethics approval was granted by the University of Adelaide Human Research Ethics Committee (HREC/HL/2025/0397). All protocols and procedures were explained to participants, and informed consent was obtained from all participants prior to participation in the study. The study was conducted from January 2026 to May 2026 at Adelaide University. This article follows the Standards for Reporting Qualitative Research (SRQR) checklist [12].

This study used an exploratory qualitative design to investigate consumers' and professionals' perspectives on integrating teledentistry into a mobile oral health application. Data were collected sequentially through an online questionnaire and semi‐structured interviews. The questionnaire collected participant demographic characteristics and preliminary insights into perceptions of mobile oral health technologies, while the interviews allowed participants to explore their experiences, opinions and attitudes in greater depth.

### 2.2 Recruitment and Sampling

A purposive sampling approach was used to recruit participants with relevant experience and knowledge regarding oral health, digital health technologies and mobile application development. Four groups were targeted: (i) parents and caregivers of children aged 4–15 years; (ii) public health professionals; (iii) software and design professionals with experience in mobile application development; and (iv) dental professionals. Eligibility criteria varied by group. Public health professionals were required to have experience in public health, paediatric health, education or health policy. Parents and caregivers were required to have at least one child aged between 4 and 15 years. Software and design professionals were required to have experience in mobile application development or digital product design. All participants were required to be aged 18 years or older.

Participants were recruited through the research team's professional networks, social media platforms and organisational contacts. Snowball sampling was also used, in which participants were encouraged to share information about the study with potentially eligible individuals in their professional or personal networks. Sample size was guided by the concept of information power rather than a predetermined target or data saturation; recruitment across the four participant groups continued until the breadth and depth of perspectives were judged sufficient to address the study aim [13].

### 2.3 Data Generation

Participants who completed the online questionnaire were then invited to take part in a semi‐structured interview to further explore their views and experiences regarding teledentistry within a mobile oral health application. Interviews were conducted by members of the research team (J.G., R.A., S.N., L.D) either in person, by telephone or via videoconferencing, depending on participant preference. Each interview was conducted by a single interviewer. The use of four different interviewers may have introduced some variation in interview style and depth of responses, although this is not considered likely to have substantially affected the overall richness of the data. Interview guides for each participant group were pilot‐tested and refined prior to data collection. Each interview guide comprised 7–14 open‐ended questions, tailored to each participant group, with follow‐up prompts (Supplementary File 1). Interviews were semi‐structured: all participants were asked the same core questions, but the order was flexible, and interviewers used prompts to follow up on issues raised by participants. Open‐ended questions such as ‘Could you see telehealth used in an oral health educational app?’ were used, and interviewers followed participants' lead with prompts. Interviews were audio‐recorded with participant consent, transcribed verbatim and de‐identified prior to analysis.

### 2.4 Data Analysis

Qualitative data were analysed using reflexive thematic analysis in an iterative process across six phases [14]. Firstly, two researchers (J.G., R.A.) familiarised themselves with the data through repeated reading of transcripts and review of audio recordings, while documenting reflections and analytic observations in a shared document.

Initial coding was undertaken by the same two researchers (J.G., R.A.), who led ongoing discussions with the wider research team to explore interpretations, challenge assumptions and deepen analytic development. The two researchers discussed codes and grouped them into broader patterns of meaning, from which preliminary themes and subthemes were developed in a collaborative process. Consistent with reflexive thematic analysis, these discussions aimed not to measure inter‐coder agreement but to deepen and challenge interpretations. Because the study brought together four distinct stakeholder groups, the interviews were analysed as a single combined dataset rather than group by group, so themes could be developed while each group's perspective remained visible within them. The authors compared patterns across parents and professionals, distinguishing points of convergence, where a view was shared across groups, from points of divergence, where a view was particular to, or contested by, a single group. Convergent patterns formed the basis of themes, and divergent perspectives were retained and reported rather than smoothed over, so that both common ground and meaningful differences between groups were represented in the findings. The team refined and finalised themes, then brought the analytical narrative together in the final step.

### 2.5 Research Reflexivity

The research team (i.e., the authors who designed the study and collected and analysed the data) comprised researchers, dental clinicians, public health professionals and parents of children within the target age range, bringing diverse professional and lived experiences to the study. These perspectives informed the study design, data collection, analysis and interpretation of findings. The research team maintained reflexivity throughout the process through reflective note‐taking and regular discussions. These discussions encouraged critical examination of assumptions and interpretations, ensuring the findings reflected participants' diverse perspectives on teledentistry within an oral health mobile application.

## 3 Results

### 3.1 Participant Characteristics

A total of 59 participants completed both the online questionnaire and the subsequent semi‐structured interview, comprising 39 women and 20 men (Table 1). Participants included 15 dental professionals, 20 public health professionals, 13 parents and caregivers and 11 software and design professionals with experience in mobile application development. Dental professionals included dental specialists, general dentists and oral health therapists representing a range of career stages and experience levels. Public health professionals comprised educators, practitioners and researchers, with many reporting more than 10 years of professional experience. Software designers were predominantly UX/UI designers, most of whom had fewer than 5 years of industry experience. Amongst parents and caregivers, most reported having one or two children, and the majority held a university degree.

**TABLE 1:** Descriptive statistics of participant groups (*n* = 59).

Participant category	Demographic characteristic	Level	*n* (%)
Dental professionals	Professional role	Dental specialist	2 (13.3)
		General dentist	9 (60.0)
		Oral health therapist	4 (26.7)
	Years of experience	≤ 5 years	6 (40.0)
		6–10 years	4 (26.7)
		11–20 years	2 (13.3)
		> 20 years	3 (20.0)
Public health professionals	Professional role	Educator	6 (30.0)
		Public health practitioner	7 (35.0)
		Public health researcher	5 (25.0)
		Other public health professional	2 (10.0)
	Years of experience	< 5 years	7 (35.0)
		6–10 years	2 (10.0)
		11–20 years	5 (25.0)
		> 20 years	6 (30.0)
Parents and caregivers	Number of children	1–2 children	12 (92.3)
		3+ children	1 (7.7)
	Highest level of education	Trade or TAFE	5 (38.5)
		University or higher	8 (61.5)
Software and design professionals with application experience	Professional role	UX/UI designer	7 (63.6)
		Other software/design professional	4 (36.4)
	Years of experience	≤ 5 years	9 (81.8)
		6–20 years	2 (18.2)

*Note:* Values are presented as *n* (%). Percentages are calculated within each participant category.

### 3.2 Thematic Analysis

Four themes were generated: (1) Acceptance of a teledentistry feature in an oral health mobile app; (2) Teledentistry in oral health mobile apps improves access and equity; (3) Preference for in‐person care: diagnostic accuracy, communication and relationships; and (4) Teledentistry is logistically challenging (Figure 1). Views on teledentistry converged across the four participant groups in several respects. Participants from all groups accepted teledentistry as a complementary feature for screening, triage and follow‐up (Theme 1 in Section 3.2.1) and saw its potential to improve access for rural, remote and otherwise isolated families (Theme 2 in Section 3.2.2). The preference for in‐person care for diagnosis and treatment (Theme 3 in Section 3.2.3) was also shared across groups and was expressed strongly by parents and public health professionals, with public health professionals additionally raising cultural safety for Aboriginal and Torres Strait Islander families. Implementation concerns (Theme 4 in Section 3.2.4) differed by group: dental professionals focused on billing, funding, clinician availability, governance and consent; parents questioned the workforce needed to staff such a service; and software and design professionals focused on the cost of building the feature.

**FIGURE 1:**
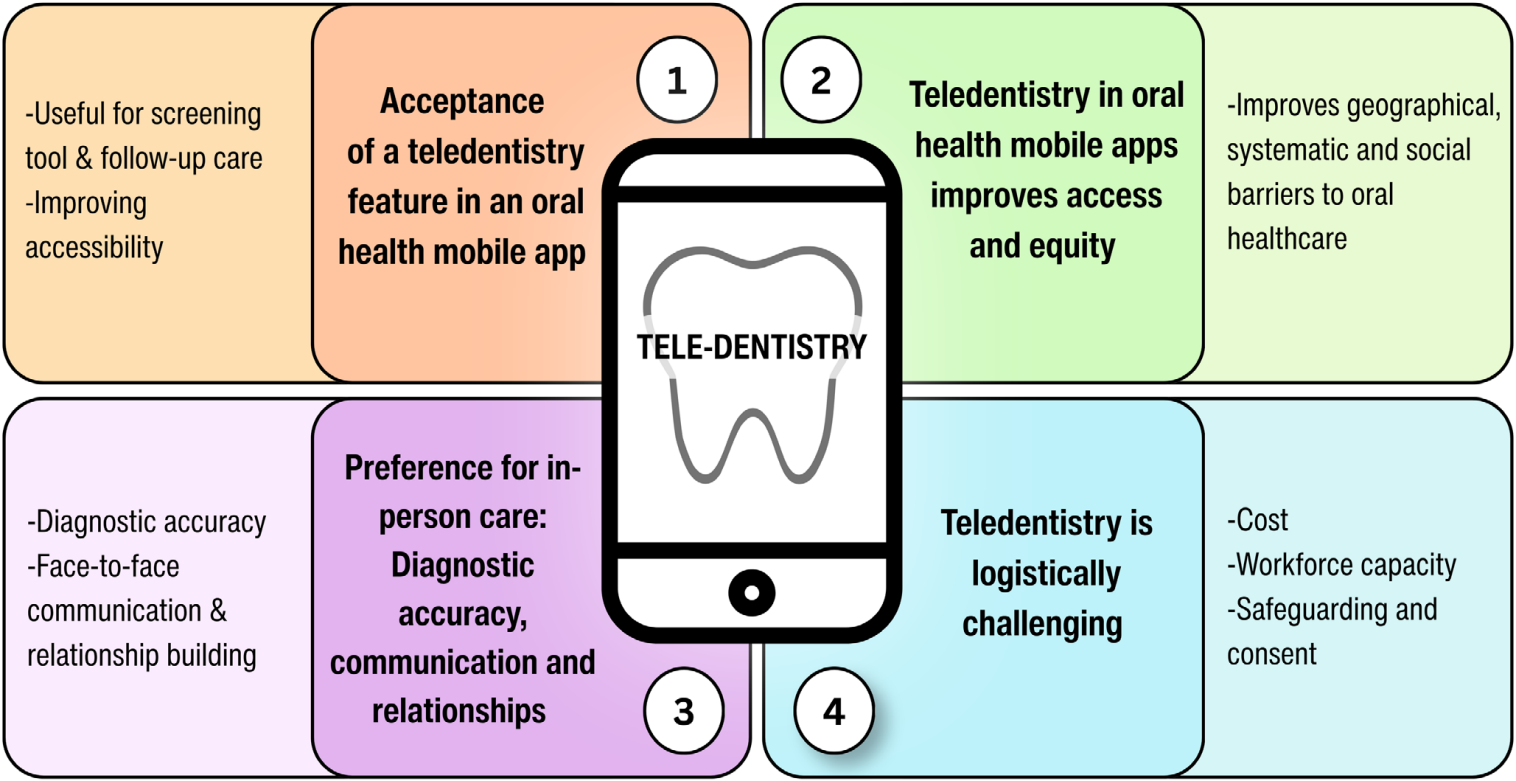
Thematic diagram. Four themes were generated: (1) Acceptance of a teledentistry feature in an oral health mobile app; (2) Teledentistry in oral health mobile apps improves access and equity; (3) Preference for in‐person care: diagnostic accuracy, communication and relationships; and (4) Teledentistry is logistically challenging.

#### 3.2.1 Theme 1: Acceptance of a Teledentistry Feature in an Oral Health Mobile App

Most participants viewed teledentistry as an acceptable and practical feature within an oral health mobile application, particularly for screening, improving accessibility and follow‐up care. Participants recognised that while dental treatment itself could not be delivered remotely, telehealth still offered value in screening for urgency and facilitating referrals or scripts: ‘the child chipped a tooth and I mean something like [teledentistry] would be great’ (Parent 17). Another parent expanded on this concept:

So definitely telehealth could be good if nothing else for screening. I mean, obviously you can't provide treatment over the telephone or over Zoom, but you can certainly, you might be able to get an indication on the urgency of treatment and then, you know, make sort of referrals. (Parent 11)

One public health professional also acknowledged the potential of telehealth to improve access to healthcare but noted that practitioners struggle with how to embed it within their practice.

Telehealth is one of those really interesting areas where, I think, that there's a bigger role than what we're currently using it for, but everyone sort of struggles with how to embed it in their practice. (Public health professional 15)

A dental professional described the current use of telehealth within the public sector, characterising it as a valuable communication tool for maintaining continuity of care through follow‐up consultations:

We actually use telehealth a lot in the public system, but we use it when we know the patient already and we saw them. So yeah, I think you're partly right that we rely on face‐to‐face first and then rely on telehealth for follow‐up. (Dental professional 41)

Building on this idea of telehealth as a tool for ongoing follow‐up, participants also felt that integrating teledentistry within the app could strengthen continuity of care by promoting accountability and sustained engagement with oral health behaviour:

… from a continuity of care perspective, it would be really good. I think it would keep parents more accountable and kids more motivated to tick things off and to have that somehow. (Parent 19)

Overall, participants demonstrated strong acceptance of teledentistry as a complementary feature within an oral health mobile application. While recognising its limitations for delivering hands‐on treatment, participants highlighted its value and potential in supporting access to care, screening and triage, improving convenience for busy parents and promoting continuity of care through ongoing engagement and accountability.

#### 3.2.2 Theme 2: Teledentistry in Oral Health Mobile Apps Improves Access and Equity

Participants perceived teledentistry within an oral health mobile app as having potential to improve access to oral healthcare, particularly for populations who experience barriers to attending face‐to‐face dental services. Discussions highlighted the perceived benefits of teledentistry for rural and remote communities, while also acknowledging that barriers to access extend beyond geography. Participants described how teledentistry could provide access for remote populations who may otherwise struggle to obtain timely dental advice. One participant highlighted how teledentistry could reduce the burden of travel, assist with urgent treatment needs and provide support in communities where access to a dentist is limited:

I think it's kind of, it's almost essential in some [remote] communities because they just can't see dentists that regularly, to have some other contact with a dentist in between to help. I guess, you could send through images, if possible, to a dentist over telehealth, and they could see. At least that way, I'd be more confident that the dentist would actually just straight up be like, ‘I can't tell from this image. I would prefer you to just go see someone’ versus, ‘yeah, it doesn't look that bad. So you should be OK for a month or two until you see someone’. (Public health professional 16)

While many participants discussed the benefits of telehealth for remote populations, others recognised that barriers to accessing oral healthcare extended beyond geographical location, noting that families in metropolitan areas may also experience societal or systemic isolation from health services:

Of those population groups, but even people in metro areas who might be sort of isolated in other ways from health practitioners. So an app that allows patients and parents, you know, kids and parents, to be able to interact with a health professional and seek advice or to, you know, use images or other data from that. I think, yeah, potentially could be quite useful. (Public health professional 15)

In addition to improving access and equity for underserved populations, participants also perceived teledentistry as having broader benefits for healthcare service delivery. Some participants felt that providing opportunities for families to seek advice remotely and to triage those needing priority screening could help reduce unnecessary appointments and ease pressures on overstretched public dental services:

I think it'll relieve a lot of pressure as well on the public health system, I can see that happening, or even private, because people can call up and enquire about certain things. (Parent 23)

Across participants' accounts, teledentistry was perceived as having strong potential to improve access to oral healthcare by addressing geographical, social and systemic barriers to care.

#### 3.2.3 Theme 3: Preference for In‐Person Care: Diagnostic Accuracy, Communication and Relationships

A concern shared across participant groups was a preference for in‐person dental care, reflecting the view that diagnostic accuracy, rich clinician‐patient communication and the trust built face‐to‐face are difficult to replicate virtually. While telehealth can enhance access in specific contexts, participants emphasised that the tactile, experiential and pain‐related dimensions of oral health often necessitate direct diagnosis and treatment. Emphasising that reliable diagnosis in dentistry often hinges on direct examination, a parent with a dental background reinforced the limits of telehealth for oral diseases and conditions, even while acknowledging its role in improving access:

Well, my personal preference is for in‐person, particularly because I'm also a clinician. But I mean, I think there's certainly a need for the availability of telehealth because for various reasons, people can't access a dentist or a dental clinician in a timely manner due to transport or cost or whatever… But ultimately, I think it's a little bit different than sort of the medical model where, you know, a GP could, you know, diagnose someone over Zoom and then write a prescription that gets sent to the pharmacist and a patient can go pick up their medication. So, with dentistry, patients need to see a clinician if they need any treatment done. (Parent 11)

Expanding on the role of in‐person assessment for diagnostic accuracy, another participant noted that clinically significant dental pain is highly subjective and may present with minimal visible signs (hence the use of radiographs), thereby limiting the diagnostic utility of video‐based assessment:

Potentially, but so much of dental is also the feeling associated with it, right? Which may not physically show much of anything, but might be incredibly painful. And so having the ability to communicate that pain threshold … in addition to something visual or scanning or whatever, right, should be taken into account. Because so many things that can happen in the mouth, like they're experiential. They're not necessarily something observable and physical, I guess. (Public health professional 18)

Building on the importance of communication and trust, another participant's perspective foregrounded cultural safety and relational continuity. For many Aboriginal and Torres Strait Islander Peoples, telehealth can feel impersonal, making face‐to‐face contact the safer, more respectful way to connect.

It's better than nothing. But I think for a lot of Aboriginal folk, it's just another barrier of thinking that they might experience racism. They just don't quite know what the [relational] connection's going to be. It's so much nicer to have a face‐to‐face meeting than a Zoom meeting, you know. (Public health professional 17)

Collectively, participants positioned in‐person dental care as essential for accurate diagnosis, effective communication and building trust, particularly when symptoms are subjective, complex or not readily visible through telehealth. While participants recognised telehealth as useful in some contexts, they viewed it as a supplementary model of care rather than a replacement for face‐to‐face dental assessment and treatment.

#### 3.2.4 Theme 4: Teledentistry Is Logistically Challenging

Participants raised various feasibility and governance concerns about embedding teledentistry within an oral health app, particularly in relation to cost, workforce capacity, billing arrangements and service oversight:

I do worry that telehealth may be a bit too costly just because from, like, my experience, I just literally ring up, like, the [dental clinic], and sometimes they'll be able to ring up the dentist straight away on the phone. I'm just trying to balance out, like, the cost against, like, having that feature as a part of your oral health app. (Software and design professional 13)

Beyond the initial cost of adding teledentistry to the app, participants also questioned the ongoing resources required to operate such a service. In particular, the workforce required to respond to calls or enquiries was identified as a consideration for the practical implementation and sustainability of telehealth within an oral health mobile app, with one parent describing it as a good idea in principle but questioning the labour and costs involved in having people available to take calls (Parent 23).

Another participant extended this concern about workforce capacity, highlighting the operational complexity of integrating dental professionals into telehealth services. In particular, questions around billing arrangements and clinician availability were seen as important barriers to implementation:

Well, I just don't know how like billings would be and like, would you just have like a dentist on call to like man the telehealth hotline? It just sounds like it will become a lot more complicated if there's a dentist there. (Dental professional 39)

These uncertainties around billing and the need for dentists to staff a telehealth line also intersected with questions about how such services would be funded within existing schemes, such as the Child Dental Benefits Schedule (CDBS), raising doubts about whether parents would ultimately be willing or able to pay for teledentistry appointments.

So there would be some sort of gap, even if the [Child Dental Benefits Schedule] covered part of it? So I think it would be useful…I even think it would be popular. Like if a parent had an extra 15 minutes in their day, they would like to be able to schedule an appointment they could take from home. But I question would they pay for it? (Dental professional 38)

These concerns about who would fund teledentistry and whether families would face out‐of‐pocket costs also raised broader questions about who should be responsible for delivering such services. In this context, another participant highlighted the importance of governance and transparency, expressing greater trust in teledentistry models delivered through official or government‐supported channels rather than private or for‐profit providers.

See, this is where I have trouble because there are services, but they're for‐profit services and some are masked as not‐for‐profit, but are actually for profit. And that's where there's a grey line that I get worried about. And there could be ulterior motives behind telehealth services that aren't through an official capacity…Like if [government dental service], for instance, had a telehealth hotline, I'd feel comfortable with that. (Dental professional 33)

Particular attention was drawn to the safeguarding requirements unique to paediatric patients, where parental involvement and consent become essential considerations in any telehealth model:

… with I guess specifically with kids, you obviously need that level of consent. So, you would need to make sure like a parent is managing that. (Dental professional 40)

Together, these concerns underscore that the feasibility of teledentistry within an oral health app extends beyond technical implementation. For such a service to be both sustainable and acceptable, professionals and consumers emphasised the need for transparent governance structures, equitable funding arrangements, adequate workforce resourcing and robust safeguarding protections, particularly when the intended users include children and families from vulnerable communities.

## 4 Discussion

This study explored professional and consumer perspectives on integrating teledentistry into mobile oral health applications for children. Overall, participants were receptive to the inclusion of a teledentistry feature and recognised its potential to improve access to oral healthcare, particularly for paediatric populations experiencing geographical, financial and mobility‐related barriers. However, participants also highlighted concerns regarding diagnostic accuracy, communication quality, clinician‐patient relationships and practical implementation challenges. These findings suggest that while teledentistry may enhance access to oral health services, participants largely viewed it as a complement to, rather than a replacement for, traditional face‐to‐face care.

Most participants were generally accepting of teledentistry features, consistent with previous research demonstrating growing acceptance of digital health technologies in healthcare [15]. This aligns with international research, where patients and healthcare professionals have identified teledentistry as an acceptable way to deliver oral health information, triage, consultation and follow‐up care, particularly when face‐to‐face services are difficult to access [1, 2]. The increasing familiarity with telehealth services following the COVID‐19 pandemic in Australia has further contributed to professionals' and consumers' willingness to engage with teledentistry, particularly when integrated within an existing mobile health platform [5]. Importantly, acceptance in a paediatric context is shaped largely by parents and caregivers, who act as gatekeepers and facilitators of a young child's care by capturing intraoral images or joining consultations on the child's behalf. Recent evidence indicates that parents and caregivers find app‐based paediatric teledentistry an acceptable and convenient option for advice, triage and follow‐up, while remaining cautious about presentations they feel require hands‐on examination [8, 9].

A prominent finding across all participant groups was the perceived ability of teledentistry to improve children's access to oral healthcare and promote greater equity in service delivery. These perceptions align closely with existing evidence demonstrating that teledentistry can reduce geographical barriers, facilitate earlier access to professional advice and improve connections between patients and oral health providers [5, 6, 16]. In Australia, where significant disparities in oral healthcare access exist between metropolitan and rural populations, teledentistry may provide an important mechanism to extend preventive oral health services and support more equitable models of care [17, 18]. Participants also highlighted the potential to embed teledentistry within routine oral health promotion activities, suggesting that mobile applications may combine education, prevention and clinical support in a single digital platform. This is supported by growing evidence indicating that mobile health applications can successfully integrate oral health education, preventive interventions and remote clinical support, thereby enhancing access to routine oral healthcare [19, 20].

Despite these perceived benefits, participants across all groups consistently emphasised that teledentistry should not replace traditional face‐to‐face dental care. Concerns regarding diagnostic accuracy were particularly prominent, with participants questioning whether photographs, video consultations and patient‐reported information could adequately support clinical decision‐making. Previous studies report similar concerns, noting that while teledentistry may be effective for screening, triage and follow‐up care, limitations remain in assessing complex oral conditions that require a comprehensive clinical examination [5, 21]. Participants also expressed concerns regarding communication quality and the potential impact of remote consultations on the development of trust and rapport between children, parents and oral health professionals [22]. These diagnostic concerns take on particular significance in children, where remote assessment depends on the quality of parent‐captured images and on a young child's cooperation during image capture. Encouragingly, paediatric evidence suggests these barriers are often surmountable for screening purposes: Parent‐ or caregiver‐operated smartphone and app‐based screening has identified dental caries in preschool and school‐aged children with accuracy and reliability comparable to in‐person visual examination [23]. This supports participants' view that teledentistry is well suited to paediatric screening, triage and monitoring, even where definitive diagnosis and treatment continue to require in‐person care. These findings suggest that participants view teledentistry as most valuable when used as a complementary service that supports, rather than substitutes for, in‐person care.

Participants further identified a range of logistical and implementation challenges that may influence the successful integration of teledentistry into mobile oral health applications. A key concern was the cost of providing teledentistry services and uncertainty regarding funding models, including who would bear the financial responsibility for consultations and whether providers would be adequately reimbursed for their time. Participants also questioned the existing oral health workforce's capacity to deliver additional telehealth services alongside current clinical demands, particularly in settings already experiencing workforce shortages. Previous telehealth and teledentistry research has also raised concerns about reimbursement, workforce sustainability and service integration [1, 5]. However, despite these implementation challenges, teledentistry has demonstrated potential economic benefits in specific contexts, with an Australian study reporting that a teledentistry model for school dental screening reduced healthcare costs by improving workforce efficiency and minimising travel and service delivery expenses [24]. To date, no research has compared these potential economic benefits with the implementation costs and service‐delivery considerations of teledentistry for oral health promotion in Australia. Safeguarding and consent were also viewed as important considerations, particularly when delivering services to children through a mobile application. Participants highlighted the need for clear processes to verify parental consent, protect patient confidentiality and ensure appropriate management of clinical information shared remotely. These findings suggest that successful teledentistry implementation depends not only on technological capability but also on sustainable funding mechanisms, workforce support and robust governance frameworks that prioritise patient safety and ethical practice.

### 4.1 Strengths and Limitations

A key strength of this study was the inclusion of multiple participant groups, including parents and caregivers, dental professionals, public health professionals and software/design professionals. Capturing multiple voices enabled exploration of diverse perspectives on integrating teledentistry into a mobile oral health application and provided insights from individuals involved in the development, delivery and use of digital oral health technologies. Furthermore, the study addresses an emerging area of research by examining perceptions of teledentistry embedded within a mobile oral health platform, an area that has received limited attention within the existing literature.

Several limitations should also be considered when interpreting the findings. Participants were recruited using purposive and snowball sampling methods, which may have resulted in the inclusion of individuals with a greater interest in oral health or digital technologies. In addition, the study did not capture the perspectives of populations who may be disproportionately affected by limited access to oral healthcare and oral health inequities, including people living in regional and remote areas and Aboriginal and Torres Strait Islander Peoples, as these groups often experience greater barriers to accessing preventive oral healthcare.

Some quotes may also reflect second‐hand accounts of Aboriginal and Torres Strait Islander Peoples' experiences, rather than perspectives expressed directly by community members themselves. While these accounts provide valuable professional insights, they may not fully capture the lived experiences, perspectives or priorities of Aboriginal and Torres Strait Islander communities.

### 4.2 Recommendations and Future Research

Recommendations arising from these findings are summarised in Table 2. Future research should explore the implementation and evaluation of teledentistry‐enabled mobile oral health applications in real‐world settings. Pilot studies examining usability, user engagement and acceptability amongst both consumers and oral health professionals would provide valuable evidence to inform application development and refinement. Further research is also needed to evaluate the clinical effectiveness and cost‐effectiveness of integrated teledentistry services, particularly for populations experiencing barriers to accessing traditional dental care.

**TABLE 2:** Summary of findings and recommendations.

Findings	Participant concern/expectation	Recommendation	Practical application
Acceptable for screening and triage.	Timely professional advice to determine the urgency of dental problems.	Integrate asynchronous and synchronous triage functions.	Allow users to submit photos and symptoms for clinician review with advice on urgency and next steps.
Valuable for follow‐up consultations.	Expected ongoing monitoring without unnecessary clinic visits.	Incorporate follow‐up consultations into the app's care pathway.	Enable scheduled virtual reviews and progress monitoring after treatment.
Perceived to improve continuity of care.	Support for maintaining engagement between appointments.	Use teledentistry to reinforce preventive care and behaviour change.	Link teleconsultations with reminders, personalised goals and preventive education.
Improve access for rural and remote communities.	Expect easier access to dental advice despite geographical barriers.	Prioritise implementation for geographically underserved populations.	Support remote consultations, image sharing and referral pathways for rural users.
Perceived to benefit socially isolated and underserved metropolitan populations.	Recognition that barriers extend beyond geography.	Ensure equitable access irrespective of location or social circumstances.	Design inclusive services that accommodate diverse social, cultural and financial needs.
Reduce unnecessary dental visits through early advice and triage.	Expect convenient access without overburdening dental services.	Embed triage pathways that direct users to the appropriate level of care.	Provide automated guidance and clinician‐supported referrals based on presenting concerns.
Preference for face‐to‐face care for diagnosis and treatment.	Expected telehealth to complement rather than replace clinical examinations.	Clearly define the scope and limitations.	Include messaging that explains when in‐person assessment is required.
Questioned diagnostic accuracy.	Expected safe, reliable assessments despite the absence of physical examination.	Develop evidence‐based protocols identifying conditions suitable for teleconsultations.	Use structured symptom checklists and clear escalation criteria for clinical review.
Importance of cultural safety.	Expectation that services be culturally safe, trustworthy and responsive to the needs of Aboriginal and Torres Strait Islander people.	Co‐design with Aboriginal and Torres Strait Islander communities to ensure cultural safety is embedded in service delivery.	Engage Aboriginal Community Controlled Health Organisations (ACCHOs) and Aboriginal and Torres Strait Islander people in development, implementation and evaluation of the app.
Concerns around costs, workforce capacity and sustainability.	Expectation of affordable, accessible services with sufficient clinician availability.	Establish sustainable funding and workforce models before implementation.	Integrate reimbursement pathways, workforce planning and service capacity into implementation planning.
Concerns around governance, trust and provider credibility.	Service delivery through trusted public or recognised healthcare organisations.	Develop transparent governance and quality assurance frameworks.	Partner with public dental services and clearly communicate provider credentials and governance arrangements.
Requirement for privacy, consent and child safeguarding.	Expect telehealth involving children to be safe and appropriately supervised.	Incorporate robust safeguarding, consent and privacy measures.	Require parental consent, secure data management and child‐specific telehealth protocols.

## 5 Conclusion

Parents, dental professionals, public health professionals and software/design professionals generally supported integrating teledentistry as a feature within an oral health mobile application and recognised its potential to improve access to oral healthcare. However, concerns relating to diagnostic accuracy, communication and implementation challenges suggest that teledentistry is best positioned as a complementary service rather than a replacement for face‐to‐face dental care. Future development should prioritise user‐centred design, clinical governance and equitable access to maximise the benefits of digital oral healthcare delivery.

## Author Contributions

J.G.: conceptualisation, methodology, formal analysis, investigation, writing—original draft, writing—review and editing, project administration. R.A.: conceptualisation, methodology, formal analysis, investigation, writing—review and editing, supervision, project administration. L.J.: resources, writing—review and editing, supervision. T.T.: methodology, formal analysis, writing—review and editing. E.K.: methodology, formal analysis, writing—review and editing. M.T.: formal analysis, investigation, resources, writing—review and editing. S.N.: conceptualisation, methodology, formal analysis, investigation, writing—review and editing, supervision, funding acquisition.

## Funding

The authors have nothing to report.

## Ethics Statement

This study protocol was reviewed and approved by the University of Adelaide Human Research Ethics Committee, The University of Adelaide (approval number HREC/HL/2025/0397M).

## Conflicts of Interest

The authors declare no conflicts of interest.

## Supporting information


**Table S1:** Parents and caregivers.
**Table S2:** Dental professionals.
**Table S3:** Public health professionals.
**Table S4:** Software and design professionals.

## Data Availability

The data that support the findings of this study are not publicly available due to ethical and privacy restrictions, as they include potentially identifiable qualitative interview data from human participants. De‐identified data may be available from the corresponding author upon reasonable request, subject to approval by the relevant ethics requirements and institutional policies.
