# Supplementary material for: Acceptability of Teledentistry in Mobile Oral Health Applications for Australian Children: A Qualitative Study
Source: Health Promot J Austr. 2026 Nov 4;38(1):e70280. doi: 10.1002/hpja.70280 (PMC13734740; doi:10.1002/hpja.70280)
Supplement: Supplementary file 1 [file HPJA-38-0-s001.docx]

**Supplementary File 1**

**Semi-structured interview guides: questions relevant to the present analysis**

Separate semi-structured interview guides were developed for each of the four participant groups as part of a broader study of mobile oral health applications for Australian children. Interviews followed completion of the online questionnaire and were conducted in person, by telephone, or via videoconference. Each guide was organised by topic, with core questions and optional probes. Interviewers asked the core questions flexibly and used probes to follow up on issues raised by participants.

**Table S1. Parents and caregivers**

| **Topic** | **Interview question** | **Probes / follow-up prompts** |
| --- | --- | --- |
| General experience with health apps | Could you tell me about your experience with using apps to support your own health or your child/ren’s health and wellbeing? | - Have you ever used apps to book telehealth appointments? Why did you use these apps? (e.g., outside of working hours, COVID-related, too sick to leave the house) - What influenced your decision to use (or not to use) health apps? (e.g., cost, language, internet access, child’s abilities) |
| Experience with oral health apps | Have you ever used apps specifically related to teeth, brushing, or oral health for your child/ren? | - If yes: Which apps have you used? How did your child/ren respond to them? - If no: Have you ever heard of oral health apps before? Would you ever consider using an oral health app (e.g., as a reminder or timer for brushing) with your child/ren? |
| Telehealth and AI | Could you see telehealth used in an oral health educational app? | - Similar to GP-based apps, would you consider booking a telehealth consult with a dentist if something was wrong? Or would you prefer to be seen in a clinic or practice? |
|  | Could you see AI as part of an oral health educational app? | - For example, could it scan a concern within the mouth and prompt on how urgently it needs a dental professional’s opinion or review? |
| Quality and credibility | What factors do you believe make an oral health app feel trustworthy and good quality for you and your child/ren? | - For example, app design, who developed the app, where the oral health information is sourced from, or who recommends the app (e.g., dentists, GPs) |

**Table S2. Dental professionals**

| **Topic** | **Interview question** | **Probes / follow-up prompts** |
| --- | --- | --- |
| Current use in practice | Can you describe how, if at all, you currently recommend or discuss mobile oral health apps with parents and children in your practice? | - If yes: How do parents and children typically respond when you introduce oral health apps during consultations? - If no: What are the main reasons you do not currently recommend or discuss oral health apps with families? What might encourage you to use them as part of your consultations? |
| Telehealth and AI | Could you see telehealth used in an oral health educational app? | - Similar to GP-based apps, could you see patients booking a telehealth consult with a dentist if something was wrong? Or do you think in-person appointments are the only viable option for dental-related problems? |
|  | Could you see AI as part of an oral health educational app? | - For example, could it scan a concern within the mouth and prompt on how urgently it needs a dental professional’s opinion or review? |
| Accessibility | How well do you think current oral health apps cater for children of different ages, literacy levels, languages, or additional needs? |  |
| Impact on workflow and clinical processes | In what ways could oral health apps support or disrupt your normal workflow in the clinic? | - How might using an app affect consultation time, communication with families, or follow-up? |
| Risk and ethics | How do issues such as data privacy, screen time, or digital access affect your willingness to recommend oral health apps to families? |  |
|  | How do commercial elements such as advertising or product promotion influence your views on recommending these apps? |  |
| Quality and credibility | How important are aspects such as who developed the app, where the information is sourced from, and which organisations endorse or recommend it? | - For example, app design, app developer, source of oral health information, who recommends the app (e.g., dentists, GPs) |

**Table S3. Public health professionals**

| **Topic** | **Interview question** | **Probes / follow-up prompts** |
| --- | --- | --- |
| Current use in programs | Can you describe how, if at all, mobile oral health apps are currently used or discussed within your programs, services, or educational settings? | - If used: Can you walk me through a recent program, service, or class where an oral health app was used or promoted, and how participants engaged with it? - If not used: What are the main reasons they are not currently used, and what would need to change for you to consider using them? - Are there particular program types (e.g., school screenings, community campaigns) where you think apps would fit best or least well? |
| Usability for communities | Which app functions would make an oral health app most usable and relevant for the communities you work with? | - For example, multi-language options, offline access, or links to local services - Are there any apps you would recommend for particular groups (e.g., people with specialised healthcare needs or disabilities, people in rural or remote areas, Indigenous or culturally and linguistically diverse communities)? |
| Integration into practice and feasibility | What practical challenges do you foresee in integrating apps into routine school, community, or public health workflows? | - For example, staffing, time, infrastructure, coordination with other resources, or whether your target population has access to devices |
| Access and inclusion | Which groups do you believe are most at risk of being excluded or disadvantaged by app-based oral health education? | - Are there strategies you consider important to make app-based interventions more inclusive (e.g., cultural tailoring, internet access, language, disability support, marginalised communities)? |
| Telehealth and AI | Could you see telehealth used in an oral health educational app? | - Similar to GP-based apps, could you see the general public booking a telehealth consult with a dentist if something was wrong? Or do you believe dental concerns are better assessed in person? |
|  | Could you see AI as part of an oral health educational app? | - For example, could it scan a concern within the mouth and prompt on how urgently it needs a dental professional’s opinion or review? |
| Risk and ethics | What concerns do you have about oral health apps as part of large-scale health promotion or school programs? | - Are there particular safeguards, policies, or approvals you believe should be in place before recommending apps at scale (e.g., in-app purchases, product branding)? |
|  | How do issues such as commercial influence, data privacy for children, or screen-time recommendations shape your view of these apps? |  |
| Quality and credibility | How important are aspects such as who developed the app, where the information is sourced from, and which organisations endorse or recommend it? | - Can you think of a health app you like, and what appeals to you about it? (e.g., an app used to book GP consultations) |

Note: The public health guide had two branches depending on whether participants currently used oral health apps in their programs. Equivalent questions asked in both branches are combined above.

**Table S4. Software and design professionals**

| **Topic** | **Interview question** | **Probes / follow-up prompts** |
| --- | --- | --- |
| Background and experience | Can you describe your experience in designing or developing health-related mobile apps, particularly those focused on children, families, or wellbeing? | - What have you learned from previous projects about what makes a health app engaging and usable for children and their caregivers? |
|  | What are your views on using apps or mHealth to support preventive oral health education and behaviour change among children and their parents/caregivers? | - Asked of participants with exposure to oral health-related apps |
| Design considerations | If you were asked to advise on a new (oral) health app, what key considerations would you bring based on your experience? | - For example, user-centred design, integration and interoperability, gamification, strict safeguards for children - Follow-up: How about for children specifically? |
| Telehealth and AI | Could you see telehealth used in an oral health educational app? | - Similar to GP-based apps, could you see the general public booking a telehealth consult with a dentist if something was wrong? |
| Quality and credibility | What factors do you believe contribute to the quality and credibility of an oral health app? | - For example, app design, app developer, source of oral health information, who recommends the app (e.g., dentists, GPs) |
|  | How important are factors such as transparency about data privacy, accessibility features, and inclusion of diverse users when you think about an app’s quality and credibility? |  |
